# Pupils' inclusion as a process of narrative interactions: tackling ADHD typification through MADIT methodology

**DOI:** 10.1186/s40359-024-01767-w

**Published:** 2024-05-21

**Authors:** Davide Bassi, Christian Moro, Luisa Orrù, Gian Piero Turchi

**Affiliations:** 1 Centro Singular de Investigación en Tecnoloxías Intelixentes (CiTIUS), Universidade de Santiago de Compostela, Santiago de Compostela, Spain; 2 University of Padua, Padova, Italy

**Keywords:** ADHD, Inclusion, Narrations, Teacher’s training, Discourse analysis

## Abstract

**Background:**

ADHD is the most common childhood neurodevelopmental disorder. The symptomatology makes the management of ADHD particularly demanding in school, so teachers’ training programs have been widely implemented. Nevertheless, these interventions could lead teachers to concentrate on the dysfunctional elements of these students, exposing them to the risk of stigmatisation. Conceptualising stigma and inclusion as narrative processes, the present study observed how teacher ADHD training texts, endorsed by the Italian government, impact on the inclusion process of students.

**Methods:**

The research analysed a corpus of *N* = 31,261 text occurrences and focused on three areas: (1) ADHD as a clinical condition; (2) the impact of ADHD characteristics in the scholastic setting; (3) interventions to manage ADHD criticalities in school settings. To observe the interactive processes fostered by the narratives under scrutiny, we used Dialogic Science and MADIT methodology, since they allow us to measure the language use modalities through an index: the Dialogical Weight (dW). The value of dW ranges between 0.1 (min) and 0.9 (max) and is linked to the potential outcomes of inclusion for students with ADHD. A low dW accounts for narratives entrenched in personal beliefs presented as absolute truths, undermining inclusion of students with ADHD. In contrast, high dW signals language interaction relying on sharable elements, able to foster social unity and diminish stigma.

**Results:**

The results yielded a critical discursive configuration, both in general and for the three distinct areas. We measured an overall Dialogical Weight of 0.4dW and, for the three areas (1) = 0.3dW; (2) = 0.3dW; (3) = 0.4dW. The analysed text does not maximise the triggering of inclusive interactions, as they rely on individual references and present one’s narrative as the sole plausible perspective: reinforcing already existing positions and exposing to the risk of stereotyping of the pupils.

**Conclusions:**

The study highlighted how the ADHD training materials analysed, focusing on a purely informational and clinical approach, lose in effectiveness with respect to generating inclusive school settings. Finally, to promote the inclusion of these pupils, elements are offered for outlining an approach based on fostering active participation by all roles involved.

**Supplementary Information:**

The online version contains supplementary material available at 10.1186/s40359-024-01767-w.

## Background

### ADHD: from the clinic to the scholastic inclusion

According to the European ADHD guidelines [1], ADHD is the most common neurodevelopmental disorder in the mental health, paediatric and primary care departments of children and adolescents.

The diagnosis (following the criteria of DSM-5) requires the presence of developmentally inappropriate levels of hyperactive-impulsive and/or inattentive symptoms for at least 6 months in different settings, causing impairments in living [2]. ADHD is mostly diagnosed in children and adolescents, but meta-analysis showed that adults present this symptomatology too, although with lower prevalence [3–5].

Despite the age of the patient, being diagnosed with ADHD is associated with impairments in social and emotional functioning, increased likelihood of injuries, premature death, suicide, convictions, criminal and at-risk behaviours [6]. Furthermore, ADHD symptomatology can affect not only the patient, but also the relatives and all those individuals who interact in various ways with him/her [7, 8]. In this regard, the specificity of the symptomatology makes the management of ADHD particularly demanding at school.

Varrasi et al. [3] illustrate how executive dysfunction hampers the ability to follow instructions, listen, and manage frustration. Metacognitive deficits impede self-management and reflection on one’s own learning and performance. Moreover, pupils with this diagnosis frequently express emotional dysregulation, complicating interpersonal relationships and further exacerbating executive and metacognitive impairments. Taken together, these ADHD symptoms lead to reduced academic success. At the same time, these factors can be at the root of critical interactions with peers and teachers within the school environment [9–12].

Pupils diagnosed with ADHD, in fact, usually have a normal level of intelligence, but they require specific changes in the teaching approach to help their learning. On this point, ADHD constitutes the most common disorder diagnosed among the learners with SN (special needs) [13]. For these reasons, training programs for teachers have been created and implemented, aimed at developing skills and strategies useful for managing the symptomatic manifestations of ADHD in social situations [14–16], thus promoting their inclusion.

Nevertheless, when teachers are informed about the basic features of ADHD, they tend to concentrate on the dysfunctional elements of these students [17–19]. This, in turn, could lead to additional stigma-related criticalities [20]: generating, for example, a lower self-esteem of these pupils [21], a worsening of the scholastic achievements [22] and producing critical pitfalls on the management of the critical issues themselves [23], such as an overreliance on pharmaceutical treatments [24]. Therefore, it follows that these teachers’ biased attitudes, beliefs, and narrations could negatively impact the inclusion process [17, 25] and how peers will be involved in including the student with ADHD [26].

These “teacher biased attitudes”, in addition, could also have critical repercussions on a clinical level. In fact, considering the absence of a specific cause, objective assessments (such as neuropsychological tests, EEG and neuroimaging) are of limited use in clarifying the diagnosis, forcing the professional to strongly rely on the collection of anamnesis [27] and accounts produced by “non-expert roles”, such as teachers. This way the narrations of these roles assume a central part in the diagnostic procedure [1, 2, 28].

From what has been argued so far, the central role played by teachers in the management of ADHD patients emerges.

From the initial diagnosis to participation in therapeutic interventions, educators are required to apply targeted strategies and knowledge to address the challenges that may arise in an academic environment, due to the behaviours exhibited by individuals diagnosed with this condition.

These criticalities concern, above all, the peer-to-peer relations, the educational performance and the respect of the rules [11, 29]. Given this, teacher’s training regarding ADHD problems have been more and more promoted and studied among the scientific community [29].

Different research, in fact, studied the effectiveness of teacher’s training. Nevertheless, they recurred to a strictly clinical point of view, i.e. observing how much the work on antecedent and consequent behaviours reduced the critical conduct (i.e. the symptoms) of pupils diagnosed with ADHD [29–31]. Even though these studies can be useful for clinical purposes, they fail in assessing how these interventions impacted on school interactions. This, in turn, leaves unanswered questions regarding, for example, the relationship between these interventions, the emergence and the consolidation of stereotypes and, thus, their impact on the inclusion process.

### Inclusion as a narrative issue

In the previous paragraph we emphasised the need to provide tools capable of observing if, how and to what extent the narratives generated by and during teachers’ training foster the emergence of stereotypes or whether they promote inclusive processes.

In this regard, drawing from Meininger [32], inclusion can be understood as a process involving people’s stories and narrations. Consequently, intervening on the processes of inclusion implies intervening on the narrative processes through which people construct their biography, and assess the extent to which these narrations can link and interact with other narrations.

De Luca Picione et al. [33], for instance, collected the narrations of students regarding their experiences of inclusion within the educational context to observe the impact on their sensemaking process. Similar research, albeit with different methodologies, has also been carried out by Savarese & Cuoco [34], Lawson et al. [35] and Hamre [36], where qualitative approaches have been used to investigate how the inclusion process is described and built through the narrations of respondents.

As much as the aforementioned studies are appropriate and pertinent to the subject of inquiry, their approach is inherently applicable only in a post-hoc manner, i.e. after the educational interventions have been implemented.

To tackle this issue, several scholars focused on the analysis of the informational material used to structure and substantiate these informational training programs. Doing so requires understanding ADHD teachers’ training textbooks, rather than static text, as potential material to build interactions with other linguistic productions. In this processual perspective, narratives’ pragmatic value depends on their potential of opening or closing dialogues with other discourses [32]. This way, adopting an “ex-ante” logic, they aim to anticipate the impact of the informational material on the audience.

Within this research frame, it is possible to include the research carried out by Freedman [37], using discourse analysis to investigate the narrations conveyed by the textbooks used for teacher training in the USA. The author emphasises how, starting from these textbooks, teachers may rely more on medical advice than on diverse teaching strategies; secondly, the policies they develop may contradict the objective of inclusive education, hindering the generation of an inclusive and diverse educational environment.

Another investigation into ADHD informational content in the USA is the study by Erlandson et al. [38]. Using Critical Discourse Analysis (CDA), the researchers identified several significant issues, including argumentative fallacies that circularly pose ADHD manifestations as causes and effects, as well as an excessive dependence on biomedical interventions.

Similarly, Te Meerman et al. [39] scrutinised ADHD information conveyed by academic textbooks, scientific articles, websites and videos. The authors employed CDA and Qualitative Content Analysis (QCA) to analyse both the content (e.g. nouns, acronyms and metaphors choices) and the possible presence of logical fallacies (e.g. circular argumentations and generalisations). This analysis revealed how informational texts can contribute to reify^1^ ADHD.

Lastly, we acknowledge the study conducted by Val Langen et al. [40], wherein 41 psychoeducational textbooks from the USA, UK, Netherlands and Hungary were examined through discourse analysis. Once identified the main themes conveyed (i.e. “definition of the diagnosis”, “causes and risk factors” and “treatments”), the authors focused on the discursive patterns used to articulate them. Across the four countries, the authors observed several internal conflicts in how ADHD was framed and contextualised, potentially leading to confusion among the audience.

Our study aligns with these previous investigations yet sets itself apart by employing a methodology that *quantifies the degree of inclusivity* rendered by the materials analysed (see “Methods” for further explanations). The introduction of a measurable index provides two primary benefits: (1) it facilitates rigorous and comparably easier evaluations across different studies; (2) it necessitates a comprehensive review of the texts under examination, ensuring that both their strengths and limitations are duly considered. Additionally, this study investigates educational materials endorsed by the Italian government, a country that is missing from the studies in this field, thereby filling a significant gap and adding a unique perspective to the existing research landscape.

## Methods

### Theoretical and methodological references

This study aims at observing how ADHD teachers’ training textbooks, endorsed by the Italian government, impact on the inclusion process of students diagnosed with this disorder. To this extent, the research is akin to the one carried out by Freedman [37] in the United States. Nonetheless, we referred to a different methodology in order to observe, in addition to the contents used, the processual dimension of the narratives proposed in these documents and to measure how much these modalities expand the range of possible interactions.

**Fig. 1:**
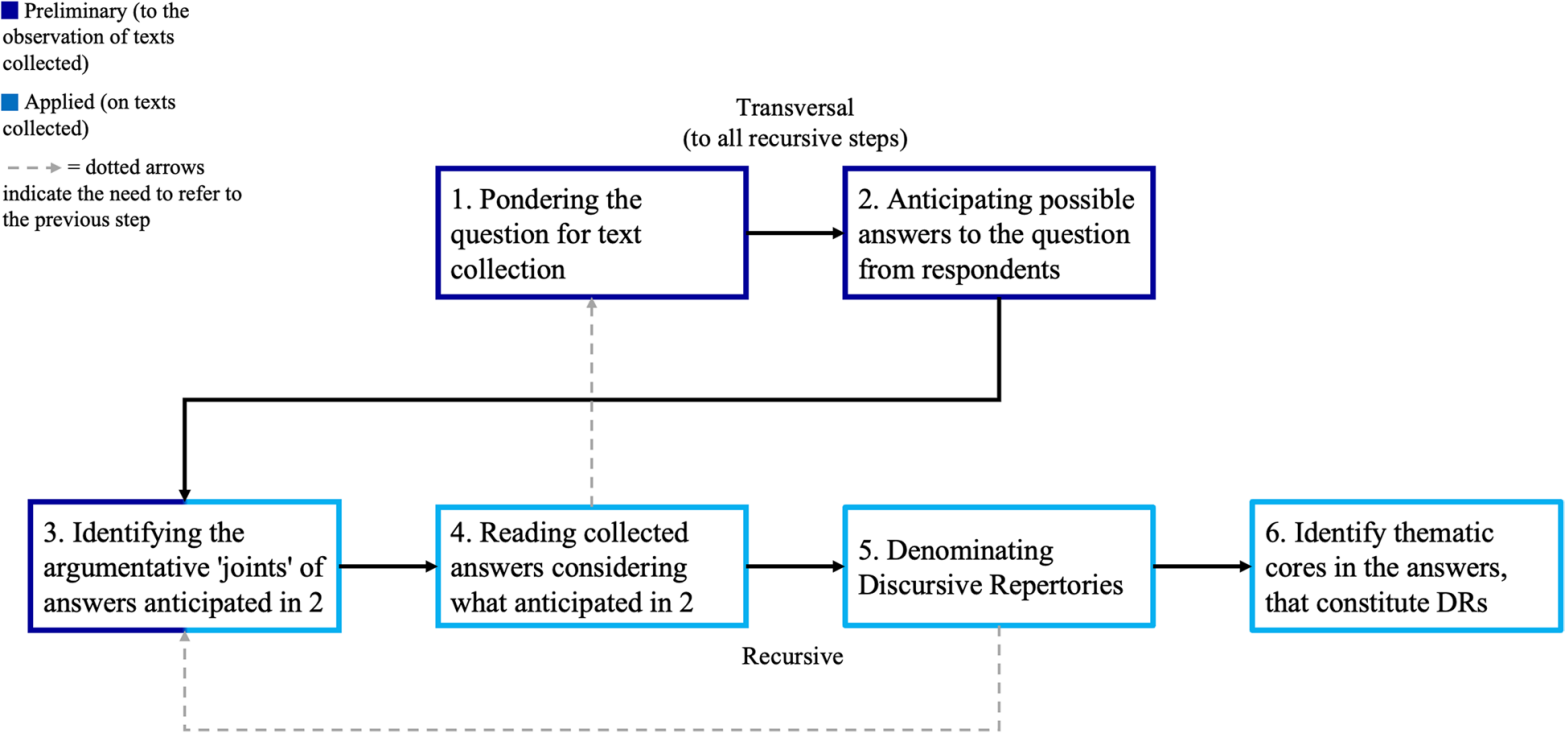
- MADIT’s procedure for text data analysis

To achieve the aforementioned objective, we drew upon the theoretical references of Dialogic Science^2^, which follows the Narrativistic Paradigm [42, 43]. The methodology employed for the text analysis is MADIT (Methodology for the Analysis of Computerised Text Data) [42, 44]. Dialogic Science and MADIT provide the researcher with, respectively, a theoretical basis and a methodological approach for describing and measuring the use of natural language. This approach is based on the encoding of 24 Discursive Repertories (DRs)^3^, each one representing a specific modality of using natural language (see Supplementary Materials 3). Humans can create countless narrative realities through language, nevertheless all these realities of sense can be described as a set of these modalities which, in turn, create a discursive configuration^4^ that can either change over time or remain static. MADIT’s procedure for text analysis comprises 6 sequential steps (Fig. 1, following the black arrows): the first two are defined as ‘transversal’ as they are performed only once. The latter four are defined as ‘recursive’ as they need to be performed on each single response text. Although the six steps are sequential, some of them − 3, 4 and 5 - demand the researcher to refer to previous ones (as specified in Fig. 1). In particular, steps 3 and 4 ask to refer to what anticipated in step 2: this is useful for the researcher to place him/herself in the role of respondents, in order to foreshadow a range of actually possible answers and ways of using language to configure the topic. In turn, this allows the analyst to be more precise and efficient in step 5 and 6 of the procedure. Differently, step 5 asks to refer to step 3: again, this is useful for the researcher to have a track of the most common possibilities of using language in relation to that topic (without excluding any of them a priori). Identifying the argumentative ‘joints’ of a text and denominating the DRs can be considered as concurrent processes in MADIT: in fact, in the same moment the researcher detects the argumentative ‘joint(s)’ of a text he/she knows how language has been used, and so to which DR matches.

According to their characteristics, the 24 Discursive Repertories have been categorised into three typologies: Stabilisation, Generative and Hybrid.

Stabilisation Repertories are discursive modalities characterised by producing absolutizing narratives by making use of personal references. Regarding the management of pupils with ADHD, thus, these narrations offer a single possible scenario. On one hand, they portray a stereotypical image of the student, which exhausts all potential narratives. On the other hand, they frame the approaches for dealing with these students as mechanical solutions that are generalizable and universally applicable, regardless of specific situations and individuals involved.

Generative Repertories, instead, consist of language use modalities that are characterised by crafting more possibilistic narratives, employing intelligible elements whose collectively shared value allow the interactants to orient themselves. These modalities, when it comes to management of pupils with ADHD, generate interactive scenarios in which all roles can make their contribution. Thus, they allow the identification of the characteristics of the situation under consideration and the generation of solutions that take into account the specific critical and strong points. Moreover, given the involvement of different roles, these discursive modalities are characterised for promoting social cohesion and assumption of responsibility.

Finally, Hybrid Repertories can take either a stabilisation or generative orientation, depending on the type of DR they are interacting with.

Thanks to the encoding of DRs, Dialogic Science provides a rigorous measure of natural language use modalities: the Dialogic Weight (dW). Each Discursive Repertory has its dW depending on its specific properties. dW can assume values between 0.1 and 0.9: the lower the dW, the more it will be characterised mainly by Stabilization Repertories; vice versa, a text with a high dW will be mainly characterised by Generative Repertories.

In conclusion, referring to the conceptualization of Meininger [32], it is possible to link the value of dW to the outcomes we can expect in terms of inclusion of pupils with ADHD. Namely, a discursive configuration with a low dW, generates a text grounded in personal theories and references which stands as a factual reality. This lessens the possible narrative plots ramification around the pupils diagnosed with ADHD, undermining the inclusion processes. Conversely, discursive configurations with high dW are indicative of language interaction modalities able to foster inclusive processes, reducing stigmatisation [42], social fragmentation and, at the same time, promoting social cohesion [44].

### Dataset and investigation protocol

We created an investigation protocol aimed at observing the text regarding three different aspects of the issue (see Table 1). To provide the most comprehensive understanding of the discursive configuration generated by the textbooks, each dimension aims at addressing a specific portion of the objective, moving from the more general description of ADHD as a clinical condition, then detailing its manifestation within the school environment and, finally, examining how this information translated into practical management strategies for ADHD school-specific critical issues. This choice is also consistent with Erlandson et al. [38], underscoring how approaching this topic transitioning from “the general to the specific” allows the researcher to contextualise its analysis. In this regard we emphasise how contextualisation is crucial to produce the most accurate anticipation about the narrative trajectories that a text may engender.

**Table 1:** Area of investigation and investigation protocol

Area of Investigation	Survey protocol question
1) ADHD as a clinical condition: features of the diagnostic label and theories used to substantiate the description.	*“How does the text describe the clinical features of attention-deficit/hyperactivity disorder?”*
2) The impact of ADHD characteristics in the scholastic setting: task execution, school performance, interactions with peers and teachers.	*“How does the text describe the manifestations in the school environment of a pupil with attention-deficit/hyperactivity disorder (how are the relationships between the pupil with ADHD and peers and with teachers described, how are the expectations, moods of teachers described, how does ADHD “impact” school performance and how the pupil approaches school demands and failures/successes)?”*
3) Interventions put in place to manage critical issues related to individuals with ADHD in school settings, as well as how the same are argued.	*“How does the text describe and argue for interventions to manage the behaviour of a pupil with ADHD in the school setting?”*

In order to gather the texts used for teacher training, we referred to the bibliography indicated in an official communication of the MIUR (Ministry of Education, University and Research) [46] in response to the increasing and reported presence in schools of pupils diagnosed with (see Supplementary Materials 1 for the complete list of the materials analysed). While we acknowledge that Italian professionals and teachers may refer to additional materials, we opted to analyse these texts as they encapsulate the institutional state-of-the-art perspective on ADHD. Consequently, we anticipate that these resources represent the most commonly used and widespread materials in the educational sector.

Prior to the use of MADIT for the text analysis of these documents, we carried out an initial “skimming” of the same by eliminating all the text excerpts that did not cover any of the three investigation areas. At the same time, the valid texts have been assigned to one of the three categories (see Table 2). Once this was done, each text was analysed according to MADIT’s procedure. To keep track also of the contents conveyed by the analysed texts, we created a list of “archipelagos of meaning”, i.e. content micro-categories generated according to the research objective (for the complete list of the “archipelagos of meaning used for the research see Supplementary Materials 2). Finally, using D.I.Ana. software [42], we automatically calculated the dW of the configuration, as well as the descriptive statistical indexes of the same.

**Table 2:** Text Sample divided for research area and text excerpts

Area of Investigation	Number of Words	Number of Text Excerpts
ADHD as a clinical condition: features of the diagnostic label and theories used to substantiate the description.	7919	325
The impact of ADHD characteristics in the scholastic setting: task execution, school performance, interactions with peers and teachers	5839	241
Interventions put in place to manage critical issues related to individuals with ADHD in school settings, as well as how the same are argued.	17,503	516
Total	31,261	1082

## Results

### First investigation area - ADHD as clinical condition

Table 3 delineates key DRs pertinent to the first investigation domain, alongside the corresponding archipelagos of meaning and their respective dW within the configuration (for the detailed results regarding all the 24 DRs, see Supplementary Materials 4a).

**Table 3:** Results for the configuration of the first investigation area

Discursive Repertory	Configuration %	Archipelagos of Meaning for specific DR	DR %
Certify Reality [CR]	32.62%	Genetic influences	42.86%
		Prognosis	42.42%
		Comorbidities	41.07%
Specification [SI]	15.69%	Relational difficulties in general	44.4%
		Neurobiological elements and functions	33%
		Behavioural Criticalities	19.72%
Confirmation [CP]	12%	Motivational elements	40%
		Relational difficulties in general	33%
		Comorbidities	16%

Dialogical Weight = 0.3 dW

As the table shows, the most used DR is “Certify Reality”^5^. This DR is characterised for creating a reality of sense that poses itself as a “matter of fact”. The data regarding the archipelagos of meaning, then, show how this way of configuring reality mainly conveys contents related to the technical features of the diagnosis (prognosis, aetiology, comorbidity, incidence and prevalence), with discursive production such as:

“*behaviour disorders necessarily have an organic aetiology”*.

The example shows how, using this discursive modality, ADHD aetiology is rooted into the organism of the person, without making explicit the criteria adopted to claim so. This way the description of the characteristics of the diagnosis is poorly intelligible, resulting in confusion for the receivers of the information as shown also by [40]. To deal with this, teachers could use these clinical elements appealing to personal interpretations, or even ignore them. This can reduce the capacity of these materials to aid teachers in adjusting their activities and approaches to accommodate the clinical characteristics of ADHD. Moreover, the configuration of the organism as the only possible “intervening dimension”, allows us to anticipate how these texts could be used to justify possible academic failures, ascribing them to a biological dimension where the teachers have no margin of intervention. In this regard, Te Meerman et al. [47] and Freedman [37] point out how the controversial but widespread characterization of ADHD as a genetic neurodevelopmental disorder may render educators and other teaching professionals inadequate, potentially compelling them to seek solutions beyond their own skills and resources. This reluctance may also precipitate in a more severe consequence wherein educators demonstrate an aversion to assuming responsibility for students with special needs [48].

The second DR in order of frequency of occurrence is the one of “Specification”^6^. This DR belongs to the Hybrid typology; thus, it cannot occur on its own, but needs to be associated with another. In this sense, its impact on the configuration will depend on the repertoire to which it will link.

Looking at the archipelagos of meaning, it is possible to see how this DR is used especially with reference to “relational issues”, “neurobiological elements” and “behavioural issues”. An example of the use of this DR can be observed in these two text excerpts:

**Table:** 

“*Different authors argue that the main deficit of the syndrome is precisely the difficulty of attention […]”* [CR]	“*[…] which manifests itself in both school/work and social situations”* [SI].

In this case, the second text excerpt is used to specify the reality of sense generated by the first one, providing more details about the circumstances where the attentional deficit emerges. Therefore, the text supports the established reality of sense derived from the initial one.

The third DR is the one of the “Confirmation”^7^. Also, this DR belongs to the Hybrid typology. Referring to the example below, one can observe the varying “support” provided by this DR, in contrast to that of the “Specification” DR. In fact, while the latter works by adding details to the configuration, the former plays a role of “reinforcement” providing textual elements that contribute to the maintenance of the reality of the sense generated as a sort of “proofs”.

**Table:** 

*“The adverse outcomes include delinquency and other antisocial behaviour and underachievement in school.”* [CR]	*“Longitudinal studies indicate that inattentive and restless behaviour is a developmental risk.”* [CP]

Finally, the observed value of dW (0.3 dW) underscores the prevalent application of stabilisation DR, leading us to conclude that the narrative construction of ADHD as a clinical condition creates a “closed” interactive scenario. From a more operative perspective, this suggests that teachers are likely to use clinical information on ADHD to sustain (or justify) the current situation, rather than to create new approaches for addressing and managing the unique challenges faced by these students (see also Te Meerman [47]).

Furthermore, given the critical role teachers play in the diagnostic process, the implicit value of the provided information can lead to subjective understandings of ADHD characteristics. Additionally, Freedman [37], analysing the diagnostic criteria for ADHD, identified the same subjectivity, thereby underscoring the importance of this issue. Relying on personal criteria, in fact, introduces substantial risks to the reliability and validity of the diagnosis, potentially leading to an increase in the incidence of false positive or false negative assessments.

### Second investigation area - ADHD in the scholastic setting

Table 4 contains the most frequent DRs and archipelagos of meaning used to generate the discursive configuration in relation to the second investigation area, as well as the dW of it (for the detailed results regarding all the 24 DRs, see Supplementary Materials 4b).

**Table 4:** Results for the configuration of the second investigation area

Discursive Repertory	Configuration %	Archipelagos of Meaning for specific DR	DR %
Certify Reality [CR]	37.34%	Criticalities in the execution of homework	58.82%
		Negative interaction between peers	47.02%
		Cognitive criticalities	33.3%
Cause of Action [CA]	22%	Evaluation and academic performance	56.25%
		Negative feelings	38.89%
		General difficulties in performing tasks	36.84%
Specification [SI]	16%	Reading-writing difficulties	28.57%
		Errors in assessment by the student	25%
		Motivational elements	22.2%

Dialogical Weight = 0.3 dW

Also in this case the most used DR is “Certify Reality”. However, precisely because of the different investigation area, it’s possible to observe how it was used to configure reality of sense regarding different themes, as the analysis of the archipelagos of meaning shows.

*“Teachers and parents report that children with ADHD seem not to listen or have their heads elsewhere when you talk to them directly”*.

Comparing this example with the one from the first area of investigation reveals that, despite differences in content, the narrative process promoted remains consistent: language usage is marked by reliance on personal references and theories, and it constructs a reality of sense characterised by absoluteness and immutability. This way a factual scenario is generated, where the ADHD student can occupy only the position of the problematic pupil. This stigmatising effect of ADHD information is particularly stressed also by Erlandsson et al. [38], highlighting how the analysed NIMH’s documents omit any mention of observable strengths and positive characteristics in the child.

The second most widely used DR in terms of frequency is “Cause of Action”. This DR belongs to the Stabilization typology too, nevertheless it is characterised by a different “logic”. In fact, it refers to an use of language that creates causalist links, such that given a certain element, another necessarily follows.

“*[…] often changes games or activities because in a short time he gets bored with what he is doing he is therefore looking for new things, for more and more exciting stimulation*:” (CA).

As the previous example shows, the “Cause of Action ‘’ DR accounts for a language use which poses boredom and the pursuit of excitement as the causes for the change in the game being played. Given this we can anticipate, on the teacher’s side, an “overwriting” of the actual reasons behind the change of activity of the pupil. The factuality and the absoluteness through which this causalist relation is posed may limit the teacher’s exploration of alternative solutions for addressing distractions, that is, to consider different narratives surrounding these challenges. Also this “overwriting” issue is corroborated by Erlandsson et al. [38], who note in their analysis that even when alternative explanations for a child’s behaviour are considered, they still pertain to the child’s internal state and thus are linked back to signs of a psychiatric disorder.

Overall, the 0.3 dW index represents these stabilisation trends. In fact, the argumentative modalities outlined above factually denote the criticalities encountered by the “student with ADHD” at school. Both concerning the challenges in task execution, behaviour and relational dimension, the dW index accounts for the promotion of narratives designed to stifle “potential movements” of the discursive process towards alternative scenarios.

### Third investigation area - scholastic management strategies for ADHD

Table 5 contains the most frequently used DR in the configuration of the third investigation area, as well as the archipelagos of meaning most often linked to these discursive modalities and the dW of the configuration (for the detailed results regarding all the 24 DRs, see Supplementary Materials 4c).

**Table 5:** Results for the configuration of the third investigation area

Discursive Repertory	Configuration %	Archipelagos of Meaning	DR %
Certify Reality [CR]	18.83%	Effectiveness	42.86%
		Teacher’s training	34.62%
		Psychoeducational interventions	29.03%
Specification [SI]	15.15%	Behavioural criticalities	21.88%
		Punishments and reprimands	17.65%
		Providing clarification of the activity	12%
Prescription [PT]	8.51%	Indication for teachers to be brief and essential	35.29%
		Cognitive-Behavioural interventions	24.14%
		Context structure	15.63%

Dialogical Weight = 0.4 dW

Also for the third investigation area, the most used DR is the one of “Certify Reality”, although with a lower percentage of occurrence if compared with the other two investigation areas. In this case, the contents most often associated with this DR are: the effectiveness of interventions, the teacher training and the psychoeducational interventions. Using the DR of “Certify Reality” to convey these contents, in turn, implies that the variety of potentially generable scenarios - with respect to intervention modalities - is exhausted by the ones offered in the texts. In this sense, despite the amount of strategies provided, we can observe how they are conveyed in a “compartmentalised way”, like in the example that follows:

“*The only way to reduce the behaviour of making noise is to ignore it actively*, *withdrawing all attention from the child*”.

The example allows us to observe how the suggested strategy, lacking a well-defined overall goal, results in a compartmentalised action aimed at addressing a specific issue of the pupil (the noise in this case). Starting from this, two possible critical pitfalls can be anticipated. The first regards the case in which the specific action will fail, leaving the teacher without any other solutions. The second one, instead, is related to the possible occurrence of criticalities not previously anticipated and for which, by consequence, specific “counteraction” have not been defined. Koutsoklenis [49] reached similar conclusions, highlighting that psychosocial training programs adhere to a “manualized” approach, which inhibits teachers from employing their unique knowledge and skills to create innovative and flexible interventions tailored to the specific needs of their pupils.

This “trend” in presenting the strategies is then supported by the contribution of the second and the third most frequent DR. The “logic” that characterises the DR of the “Specification” has already been discussed. Focusing thus on the DR of the “Prescription”^8^ we highlight how also this DR belongs to the Hybrid typology. It follows that its contribution to the configuration will depend on the other DRs composing the discursive configuration. In this sense, the prescriptions outlined in the analysed texts support the depicted scenario by dictating the actions that teachers are expected to follow.

**Table:** 

*“It is important to allow children to practise the skills until they perform them appropriately.”* (CR)	*“Specific feedback and modelling should be provided by the teacher.”* (PT)

In the example, the first text excerpt establishes that giving time for the child to practise skills is fundamental for managing critical characteristics of pupils with ADHD. Starting from this, the second excerpt works in a supportive way with respect to the first, more precisely, by defining the role of the teacher as the one in charge of providing the pupil with specific feedback.

Given the Hybrid nature of the “Prescription” DR, it contributes to enhance narrative’s “malleability”, particularly by getting into the specific definition of teacher’s role functions (e.g. offering specific feedback). However, in the observed stabilisation configuration, the full inclusive potential of this excerpt is somewhat impeded by its link with CR’s utilisation of personal references. In the example, for instance, the implicit value of “*appropriately*” leads the interactive management back to the subjective interpretation of this element. Concluding, coherently with the value of dW measured (0.4 dW), despite the lower percentage occurrence of Stabilization DR, the third investigation area is also oriented towards the generation of a reality of sense posed in terms of unicity and immutability. This data allows us to prefigure the criticalities described above when discussing the pitfalls deriving from the use of the DR of “Certify Reality” in relation to the third investigation area.

### General configuration

Table 6 contains the most frequently used DR in the whole configuration, as well as the archipelagos of meaning most often linked to these discursive modalities and the dW of the general configuration (for the detailed results regarding all the 24 DRs, see Supplementary Material 4d).

**Table 6:** Results for the general configuration

Discursive Repertory	Configuration %	Archipelagos of Meaning	DR %
Certify Reality [CR]	27.10%	Negative interactions between peers	39.54%
		Prognosis	39.47%
		Behavioural Criticalities	31.37%
Specification [SI]	15.26%	Negative interactions between peers	23.26%
		Elements of demotivation	20.69%
		Behavioural Criticalities	18.95%
Cause of Action [CA]	8.51%	Relational difficulties in general	54.55%
		School evaluations and performance	45%
		General difficulties in performing tasks	37.50%
Possibility [PS]	8.51%	Problems in reviewing work	22.22%
		Teacher-pupil interaction difficulty	20%
		Psychoeducational interventions	16.13%

Dialogical Weight = 0.4 dW

As we can see from the table, and according to the previously described results, the most used DR in the general configuration is “Certify Reality”, which, among the stabilisation DRs, is the one with lowest dW, indicating its strong impact in creating immutable and factual realities.

The general configuration repeats quite consistently the results discussed for the previous survey areas, with “Specification” and “Cause of Action” as, namely, the second and the third most frequent repertoires. The DR of the “Cause of Action”, however, share the same percentage of the hybrid DR of “Possibility”^9^. Focusing on the latter, it is characterised by generating a reality of sense posed in terms of uncertainty and possibility. In this sense it could be useful to break the stabilisation “coherence” of the configuration. Nevertheless, this DR, lacking a base of shared and third elements, offers no guarantee regarding the impact it will have with respect to the discursive process in which it is used. Belonging to “Hybrid” typology, it will assume a generative or a maintenance valence depending on its interaction with the other repertoires present in the discursive configuration. Considering the prevalent use of stabilisation DR, it can be anticipated that the uncertain reality of sense produced by the “Possibility” DR will recede and be integrated into the narratives created through DRs like “Certify Reality”.

Concluding, referring to the data presented in Supplementary Material 4d, we can observe how the general configuration is composed also by Generative DRs, such as the one of the “Description”^10^. This DR is characterised for building narrations based on third elements, which are commonly intelligible for the interactants. Thanks to this, “Description” is the most generative DR and it is able to maximise the possibility for narrations to “connect” and interact within each other, generating new possible and unpredicted scenarios. An example of this DR is the following text excerpt:

“*While doing homework and in-class tests, the student is concerned about the amount of exercises to be done, keeps checking how much is left to the end but fails to plan the execution of the activity.*

*The student struggles to compile a hierarchy of what is most important and where to start.”* (DE).

As we can observe from the example, the narration is characterised by portraying a widely recognizable scenario. In fact, using the “Description” DR, implies the use of elements whose value is made explicit and shared. Building the narration without connoting it with any personal judgement or opinion allows the generation of a narrative that all involved roles can use as a common reference. This, in turn, increases the possibility for each role involved (from the teacher, to the parent and the student) to provide its contributions to the interaction, promoting, between the same, assumption of responsibility and social cohesion.

Concluding, the total dW of the configuration (0.4 dW), allows us to assert how, despite the presence of Generative DR, the overall narration of the analysed material is more directed to the generation of a unique reality, which will tend to keep itself the same.

## Discussion

Given the results described above, we will now focus on the impact of the examined discursive language use modality on the inclusion process of pupils with ADHD in the school context.

Referring to the investigation area examining the description of ADHD as a clinical condition, the analysis returned us a discursive configuration whose elements are conveyed through personal references and posed in terms of absoluteness. These findings align with the analysis conducted by Freedman and Honkasilta [50] on the ADHD diagnostic descriptions within DSM-5 and ICD-10, which revealed a prevalent use of subjective language, potentially promoting the maintenance of the status quo of school interactive scenarios. Moreover, considering the use of this subjective language it is possible to anticipate that the training material would be used by teachers, based on their personal theories. Taking as an example the following text excerpt “*The child with ADHD manifests continuous agitation, difficulty in sitting and staying still in place”* (CR), it is possible to anticipate that the teacher will consider a certain behaviour as a “manifestation of agitation” on the base of his or her personal theory about what a “manifestation of agitation” is. Similar anticipation could be made regarding the “difficulties in sitting still”: pupil’s behaviour would be justified on the basis of his/her diagnosis. These results mirror and corroborate those of Te Meerman [39], which show how the description of ADHD made by textbooks, articles, and online materials contributes to reifying this diagnosis, with consequent repercussions in terms of stigmatisation.

In addition to these stigmatising consequences, considering the clinical dimension of ADHD, we highlight how these personal interpretations of the diagnosis could lead to different criticalities regarding the diagnostic process: framing non-pathological behaviours as such and vice versa.

These conclusions are in line with the ones reached by Kennerley et al. [51], who found how a lack of clarity over what each anchor point means on rating scales (e.g., one person may interpret “often” meaning daily whereas another may consider “often” to be twice a week) could negatively impact on the agreement between clinicians, parents and teacher judgements.

Given this, it is possible to assert how, in the drafting of upcoming training materials, special care should be spent to use more descriptive elements, to assist the concordance between raters using the same scale (such as “once a month” instead of “rarely”, “once a week” instead of “sometimes”, “daily” instead of “often”, etc.).

The urge to adopt more efficient diagnostic procedures is then corroborated also by the results regarding the second investigation area, characterised by a preponderant use of the DR “Certify Reality”. This implies that the narrations regarding the pupils diagnosed with ADHD are hardly changeable, i.e. the criticalities of the student are posed as immutable elements, rooted in the specificity of the diagnosis: reified, adopting the terminology of Te Meerman [39]. These narrations work generating and consolidating a stereotypical picture of the pupil diagnosed with ADHD, by virtue of which any possible new criticality is traced back to the diagnosis.

Moreover, the second most widely used DR is the “Cause of Action”. As said in the previous section, this DR is characterised for creating a causal link between two elements, as in the example “*their learning pathway is greatly hindered by clinical characteristics”* (CA). Through this DR, ADHD difficulties are linked, in a causal relationship, to the clinical characteristics. The observation of this language use in the configuration, in turn, allows us to anticipate the possibility for the teacher to use these rhetorics as a justification for the failures of the student: ending up by feeding and cementing stigmatising and stereotypical narrations.

Furthermore, the potential for generating alternative narratives about a student’s critical issues, including the reasons behind poor performance, is diminished by supplying elements that facilitate the creation of justifications by teachers and educational authorities. Thus, teachers are provided with a predefined and scientifically and institutionally endorsed narrative framework within which to contextualise their interactions with students diagnosed with ADHD.

Also these results find correspondence with previous other research in the field; Metzger & Hamilton [52], for instance, argued about the double-edged role played by the diagnosis for children and their families: on one hand it can provide access to special assistance and resources in the school but, on the other, may activate teachers’ negative stereotypes about diagnosed students.

Hence, it is possible to state that the characteristics of the pupils with ADHD, as well the clinical peculiarities of this disorder, should be conveyed in a “target-oriented way” rather than in a notional and causalist way (which, as we showed, generates, respectively, an implicit and personal understanding of the information, and the use of them in justificatory terms) (see also [50]).

These considerations lead us to the analysis regarding the last investigation area. We observed how, despite the variety of the DRs used, the dW is more oriented towards the stabilisation endpoint (0.4 dW). The procedures for managing the criticalities related to pupils with ADHD, are mainly proposed as a list of different strategies unrelated to each other, through which to manage pupils. This observation is also supported by the frequent use of the repertory of “Certify Reality” to convey content about the “effectiveness of the intervention” (42.86% of the cases). This repertory places the effectiveness of the interventions as certain, without providing third-party, commonly recognizable elements to realise it.

Configuring the intervention in a “follow the book way” (see also [49]) could potentially reduce the care outline for these pupils by following a logic of “given X problem, then apply Y intervention”.

This could lead to critical pragmatic fallout if the strategies are ineffective, or in case the student generates critical behaviours not previously covered. Providing guidance according to the logic of “Certify Reality,” interactors are not involved in the process of strategy generation, missing the opportunity to develop the necessary skills to identify new strategies in the face of the emergence of previously unanticipated problematic scenarios. At the same time, teachers’ “situated” and “role-specific” knowledge is overshadowed by a predominantly top-down approach, which consequently fails to accommodate the unique characteristics of pupils and classroom settings [36, 49].

These results are consistent with the ones of the other investigation areas and are also matched with the ones of Ward et al. [29], who observed training interventions on teachers to be effective in increasing their knowledge about the disorder (i.e. acquiring the contents) but find poor evidence about the effectiveness of these trainings in modifying the behaviour of the pupils.

To tackle these criticalities, a useful insight offered by Dialogic Science is to structure both the trainings and the materials of them following the “Targeting”^11^ DR’s logic, such as the one of the following text excerpt:

*“In order to better emphasise to the child the importance of good management of the material without stigmatising its ineffectiveness and to provide more opportunities for positive shaping by peers as well, it might be useful to introduce part of the procedure for the whole class” [TG]*.

Apart from the specific contents conveyed in the text, the example shows the main features of this DR: the strategy (i.e. “introducing part of the procedure for the whole class”) is both linked to a target (i.e. the first part of the sentence) and conveyed in a possibilistic way. The use of this language’s modality generates a third-party element (the target) that can be used by the teacher as a common reference to generate other narrations other than the one proposed.

This DR, especially when the already proposed strategies have failed, promotes the generation of the question “how the target could be pursued?”. This question can be usefully employed as reference both to create new strategies, as well as a starting point to involve also other roles in the process. In this way, the generation of new narrations is promoted and, consequently, stereotypes and stigma can be tackled.

Finally, we acknowledge the following limitations to the present study. Firstly, this study’s scope is limited by its sample size: other studies exploring special education textbooks have employed more extensive sample sizes [53]. A larger dataset could provide a wider perspective on the overarching orientation of the Italian special education textbooks, for example extending the study to the textbooks used by psychologists, psychiatrists or general physicians. Additionally [40], comparison of psychoeducational material of six different countries revealed thematic and discursive differences. Given this, future research should aim at using MADIT in a cross-country perspective. As demonstrated in [41], cultural background not only influences the content of narratives but also affects the modes of their conveyance. In this regard, we emphasise that the measurement index (Dialogic Weight) provided by MADIT would greatly facilitate these comparative efforts. Indeed, it could serve as a standardised reference across studies, enabling researchers to systematically assess and compare the influence of cultural backgrounds on the discursive modalities of psychoeducational materials.

Additionally, the application of computational methods can significantly enhance the analysis of large volumes of psychoeducational materials. In particular, deep learning BERT-based models have demonstrated effectiveness in detecting Discursive Repertories annotated through MADIT methodology [54, 55]. Such technological advancements underscore the potential for more sophisticated and extensive research in the field.

Moreover, the mere inclusion of a textbook in a curriculum does not guarantee the same use of its content in the different real-life contexts. For this reason, future studies should address the issue of exploring and understanding how the discourses embedded in the analysed textbooks engage with the discursive productions exerted by the educators and by the students as well. In this context, it would be worthwhile to conduct a comparative analysis of the interactions facilitated by biomedical-based materials currently endorsed by national health institutions and more critical, stigma-conscious resources such as [56, 57].

## Conclusions

The present study, starting from the description of ADHD syndrome, showed how the peculiarities of this diagnosis can generate critical pitfalls, especially in the academic environment. We then discussed the role of teachers both in promoting the social inclusion of these pupils, but also in interacting with clinical roles to produce the diagnosis and to deliver the treatment.

Focusing on the process of social inclusion, we presented and adopted the vision proposed by Meininger [32], which conceptualises social inclusion as a narrative process.

Accordingly, we carried out a textual analysis of the materials utilised in Italy for educating teachers about the characteristics of pupils with ADHD and the strategies for addressing their challenges to facilitate their inclusion in the classroom.

In doing so, we referred to Dialogic Science and MADIT, which allowed us to observe and measure the processual dimension generated by the language use modalities adopted in the textbooks.

The results returned us a critical discursive configuration, regarding both the description of the pupil with ADHD and the prescriptions to deal with his/her difficulties. We showed how the discursive modalities adopted to convey this information are characterised using unshared elements which, in turn, promote their use by the teachers following personal theories and beliefs.

In this sense it can be said that this material does not maximally promote the generation of inclusive interaction: although at a content and information level it provides sufficiently precise elements, it is not equally adequate in training teachers on how to use these elements to interact with pupils with ADHD while fully promoting their inclusion at school. Relying on personal references and posing one’s own narration as the only possible one, not only promotes the maintenance of one’s own positions, but also increases the possibility of the onset of controversial scenarios: where the different narrations, instead of interacting to generate something new and third, are used to prove their correctness against each other [44]. Moreover, absolutizing the generated scenario, the diagnosis of ADHD is posed as the category through which all the narrations regarding the pupil are “read”.

Considering how such narratives could then be embraced by teachers within the educational setting, and considering Meininger’s [32] conceptualization of inclusion, it becomes apparent that, as a major repercussion, these discursive practices obstruct the weaving together of narrative threads, thereby fostering stigmatisation.

In the face of this, we concluded the article proposing and discussing a possible discursive architecture to employ in teachers’ training: which could maximise the interactions between the different roles involved in handling issues connected to the pupils with ADHD. The main implication deriving from the results of our research is that teachers’ training material primarily needs to be consulted with a critical and attentive eye, translating the information into adequate interactive modalities for dealing with pupils with ADHD. This pending (as a further implication) a perhaps required revision of the same material: in fact, we contended how these textbooks ought not furnish the reader with a “final technical definition” of “what a pupil with ADHD is”, “why they are this way,” and “how to manage their difficulties”. Instead, it should supply him/her with practical and understandable resources aimed at facilitating and guiding interactions with other stakeholders involved. This way, in addition to the informative value, the training with the teachers would be enriched by an interactive one, since they provide the participants with the opportunity to offer their contributions.

This perspective, on one side, agrees with the vision of inclusion promoted by Meininger [32] and, on the other side, it is consistent with the results of the work of Flavian & Uziely [17], who showed how as teachers actively model inclusion, this helps peers of pupils with ADHD to do the same, thus developing a spread inclusive learning environment.

Concluding, we suggest that inclusive training should primarily hinge on developing interaction management skills. This orientation aims to promote an active involvement of all the school roles, thereby fostering the idea that teachers should not become mere experts on ADHD diagnosis, but rather architects of the school community.

### Supplementary Information

Supplementary Material 1.

## Data Availability

The data that support the findings of this study are available from the corresponding author, [DB], upon reasonable request.

## Footnotes

1 A socio-linguistic process in which a concept is represented as a thing existing on its own. See Te Meerman et al. [39] for additional information on this concept.

2 *“Science that has as its object of knowledge the use of the symbolic units that compose ordinary language, which gives form to discursive configurations; [it is] that cognitive apparatus that formalises the dialogical process (or discursive process)”* [41].

3 “*A finite mode of constructing reality, linguistically understood, with pragmatic value, which groups together even more enunciated (called “archipelagos of meaning”), articulated in concatenated sentences and diffused with a value of assertion of truth, aimed at generating (building)/maintaining a narrative coherence*” [45].

4 “*Discursive configuration is defined as the particular valency that elements of ordinary language (the symbolic units) manifest when generating a discursive space configured by the interactions between the different and therefore distinguishable constructions of the sense of reality”* [45].

5 *“Discursive modality that configures reality by stating a clear, certain and unalterable state of things. The possibility of transformation is unforeseen for this reality”* (see Supplementary Materials 3 and [41]).

6 *“Discursive modality that configures reality by providing a generation or maintenance of an explicit and detailed description regarding the configuration it is associated with, limiting its range of application to what is expressed”* (see Supplementary Materials 3 and [42]).

7 “*Discursive modality that configures reality by validating and supporting what expressed through the Repertory to which it relates*” (see Supplementary Materials 3 and [41]).

8 *“Discursive modality that configures reality as orders/directions given by a third “point of view” position compared to the narrator’s one. Establishes rules and/or objectives and/or roles to follow, in terms of what one “has to do” or “has not to do”. The argumentation acquires a structure founded on a relation of necessity set by a part of the text”* (see Supplementary Materials 3 and [41]).

9 *“Discursive modality that configures reality by using one’s own and exclusive criteria as the only argumentative foundation, without making them explicit and describing them in order make them shared. It configures reality in probabilistic, possibilistic and uncertain terms”* (see Supplementary Materials 3 and [41]).

10 *“Discursive modality that configures reality as a common heritage that does not belong exclusively to any narrator and it needs everyone’s contribution to be maintained. It configures a current or past reality as if the narrator were responding to a question starting with “how” instead of “why”* (see Supplementary Materials 3 and [41]).

11 Defined as *“Discursive modality that configures reality in order to set an objective/purpose/goal to another part of the text, defining actions, strategies, interventions, etc. Enables the triggering of a discursive configuration aimed at the pursuit of the defined objective/purpose/goal and, in this way, generating modalities belonging to the generative class and of maximum generative impact”* (see Supplementary Materials 3 and [41]).
