# Supplementary material for: Pupils' inclusion as a process of narrative interactions: tackling ADHD typification through MADIT methodology
Source: BMC Psychol. 2024 May 21;12:281. doi: 10.1186/s40359-024-01767-w (PMC11106906; doi:10.1186/s40359-024-01767-w)

**Supplementary Materials**

**Supplementary Material 1 – Analyzed Bibliography**

AAVV (2004) European clinical guidelines for hyperkinetic disorder – first upgrade. European clinical guidelines for hyperkinetic disorder -- first upgrade*. European child & adolescent psychiatry, 13 Suppl 1, I7–I30.* [*https://doi.org/10.1007/s00787-004-1002-x*](https://doi.org/10.1007/s00787-004-1002-x)

AAVV (1993) *Che cos’è l’ADHD*. Retrieved from ISS. Istituto Superiore di Sanità*:* [*http://old.iss.it/binary/impa/cont/FAQ11%20fase%20due%20pdf.1121414712.pdf*](http://old.iss.it/binary/impa/cont/FAQ11%20fase%20due%20pdf.1121414712.pdf)

AIDAI. (2018). *Bambini con disattenzione e iperattività. Note per gli insegnanti.* Retrieved from AIDAI. Associazione Italiana per i Disturbi dell'Attenzione e Iperattività: <https://www.aidaiassociazione.com/wp-content/uploads/2018/12/OPUSCOLO_SCUOLA_2015.pdf>

AIDAI (2018) *1^ lezione "Le abilità di autoregolazione cognitiva e comportamentale nell'alunno"*. Retrieved from AIDAI. Associazione Italiana per i Disturbi dell’Attenzione e Iperattività: [https://www.aidaiassociazione.com/wp- content/uploads/2018/12/Dispense_Insegnanti.pdf](https://www.aidaiassociazione.com/wp-%20content/uploads/2018/12/Dispense_Insegnanti.pdf)

AIFA, AIDAI, AIRIPA, SINPIA *Indicazioni per insegnanti di bambini e ragazzi con ADHD*. Retrived from ISS. Istituto Superiore di Sanità: [http://old.iss.it/binary/adhd/cont/Indicazioni_insegnanti_di_bambini_ragazzi_A DHD_2_.pdf](http://old.iss.it/binary/adhd/cont/Indicazioni_insegnanti_di_bambini_ragazzi_A%20DHD_2_.pdf)

AIFA (2008) *Strategie scolastiche che permettono di mantenere l’attenzione e l’attività dell’alunno ADHD entro limiti accettabili*. Retrieved from: AIFA Onlus. Associazione Italiana Famiglie ADHD:<http://www.aifaonlus.it/index.php?option=com_docman&task=doc_details&gid=143&Itemid=121>

Chiarenza A. G., Bianchi E., Marzocchi G. M. (2018*) Linee guida per il trattamento cognitivo comportamentale dei disturbi da deficit dell’attenzione con iperattività (ADHD)*. Retrieved from AIDAI. Associazione Italiana per i Disturbi dell'Attenzione e Iperattività: [https://www.aidaiassociazione.com/wp- content/uploads/2018/12/Linee_guida_ADHD.pdf](https://www.aidaiassociazione.com/wp-%20content/uploads/2018/12/Linee_guida_ADHD.pdf)

Erickson (2013) *ADHD a scuola Strategie efficaci per gli insegnanti - LE GUIDE ERICKSON.* Trento. Erickson.

ISS, AIFA (2007) *Protocollo diagnostico e terapeutico della sindrome da iperattività e deficit di attenzione per il registro nazionale ADHD.* Retrieved from ISS. Istituto Superiore di Sanità: <http://old.iss.it/binary/adhd/cont/Protocollo%20diagnostico%20ADHD%20020507.1178184452.pdf>

SINPIA (2002) *Linee guida per la diagnosi e la terapia farmacologica del Disturbo da Deficit Attentivo con Iperattività (ADHD) in età evolutiva*. Retrieved from AIDAI. Associazione Italiana per i Disturbi dell'Attenzione e Iperattività: <https://www.aidaiassociazione.com/wp-content/uploads/2018/12/Linee_guida_SINPIA_Diagnosi.pdf>

**Supplementary Material 2 – Archipelago of Meaning**

| **Archipelago of Meaning (ENG)** | **Archipelago of Meaning (ITA)** |
| --- | --- |
| Other elements and characteristics of the diagnosis | Altri elementi e caratteristiche della diagnosi |
| Other assessment errors on the part of the student | Altri errori di valutazione da parte dell’alunno |
| Other | Altro |
| Attracting attention | Attirare l'attenzione |
| Involvement of the pupil in the process of intervention construction | Coinvolgimento dell'alunno nel processo di costruzione dell'intervento |
| Parental involvement in the construction of the intervention plan | Coinvolgimento dei genitori nella costruzione del piano di intervento |
| Comorbidity | Comorbilità |
| Understanding of deliverables | Comprensione delle consegne |
| Correct execution of activities | Corretta esecuzione delle attività |
| Teacher-pupil communication difficulties | Difficoltà comunicazione insegante alunno |
| General difficulties in task execution | Difficoltà generali nell'esecuzione dei compiti |
| Difficulties in reading-writing and calculation | Difficoltà in letto-scrittura e calcolo |
| Difficulties in teacher-pupil interaction | Difficoltà interazione insegnante alunno |
| Difficulty in considering outcomes | Difficoltà nella considerazione degli esiti |
| Difficulty in planning the activity | Difficoltà nella pianificazione dell'attività |
| General relational difficulties | Difficoltà relazionali in generale |
| General difficulties in performing tasks | Difficoltò generali nell'esecuzione dei compiti |
| Disorder of school materials | Disordine materiale scolastico |
| Effectiveness | Efficacia |
| Elements of demotivation | Elementi di demotivazione |
| Elements of motivation | Elementi di motivazione |
| Neurobiological elements and features | Elementi e funzionalità neurobiologiche |
| Misconceptions of teachers | Errate credenze degli insegnanti |
| Sketchy execution of tasks | Esecuzione sommaria dei compiti |
| Onset | Esordio |
| Etiology | Eziologia |
| Fostering the use of computer media | Favorire l’uso dei mezzi informatici |
| Fostering the ability to plan and anticipate | Favorire la capacità di pianificazione e anticipazione |
| Teacher training | Formazione degli insegnanti |
| Providing feedback in general | Fornire feedback in generale |
| Providing positive feedback | Fornire feedback positivi |
| Providing clarification and clarification on the activity | Fornire precisazioni e chiarificazioni sull’attività |
| Game | Gioco |
| Incidence and prevalence | Incidenza e prevalenza |
| Indication for teachers to be quick or essential | Indicazione per gli insegnanti di essere rapidi o essenziali |
| Environmental influences | Influenze ambientali |
| Genetic influences | Influenze genetiche |
| Teaching problem-solving strategies | Insegnamento strategie di problem-solving |
| Negative peer interactions | Interazioni negative fra pari |
| Positive peer interactions | Interazioni positive fra pari |
| Pharmacological interventions | Interventi Farmacologici |
| Psychoeducational Interventions | Interventi Psicoeducativi |
| Cognitive-Behavioral Intervention | Intervento Cognitivo-Comportamentale |
| Ineffective modes of intervention | Modalità inefficaci di intervento |
| Ineffective modes of communicating delivery | Modalità inefficaci di comunicazione delle consegne |
| Observing and monitoring the student | Osservare e controllare l’alunno |
| Cognitive issues | Problematiche cognitive |
| Behavioral issues | Problematiche comportamentali |
| Relational problems in general | Problematiche relazionali in genere |
| Problems related to homework | Problemi legati ai compiti per casa |
| Problems in reviewing work done | Problemi nella revisione del lavoro svolto |
| Prognosis | Prognosi |
| Promotion of self-control | Promozione dell'autocontrollo |
| Punishments and reprimands | Punizioni e rimproveri |
| Adjusting peer contacts | Regolazione contatti fra pari |
| Seeking stimulation | Ricerca di stimolazioni |
| Establishing and Respecting rules | Stabilire e Stabilire e Rispettare le regole |
| Negative feelings | Sentimenti negativi |
| Negative feelings of teachers | Sentimenti negativi dei docenti |
| Structure of the context | Struttura del contesto |
| Structure of the activity | Struttura dell'attività |
| Encourage the use of computer media | Favorire l’uso dei mezzi informatici |
| Negative evaluations and academic performance | Valutazioni e rendimento scolastico negativi |

**Supplementary Material 3 – Periodic and Semi-radial Table of Discursive Repertories – Glossary**

| **I level** | |
| --- | --- |
| **Certify Reality – CR**  (Stabilisation) | Discursive modality that configures reality by stating a clear, certain and unalterable state of things. The possibility of transformation is unforeseen for this reality. |
| **Description – DS**  (Generative) | Discursive modality that configures reality as a common heritage that does not belong exclusively to any narrator and it needs everyone’s contribution to be maintained. It configures a current or past reality as if the narrator were responding to a question starting with “how” instead of “why” |
| **II level** | |
| **Specification – SI**  (Hybrid) | Discursive modality that configures reality by providing a generation or Stabilisation of an explicit and detailed description regarding the configuration it is associated with, limiting its range of application to what is expressed. |
| **Possibility – PS**  (Hybrid) | Discursive modality that configures reality by using one’s own and exclusive criteria as the only argumentative foundation, without making them explicit and describing them in order make them shared. It configures reality in probabilistic, possibilistic and uncertain terms. |
| **III level** | |
| **Opinion – OI**  (Stabilisation) | Discursive modality that configures reality by making explicit that the contents are valid and delimited within the narrator's own and exclusive perspective. |
| **Targeting – TG**  (Generative) | Discursive modality that configures reality in order to set an objective/purpose/goal to another part of the text, defining actions, strategies, interventions, etc. Enables the triggering of a discursive configuration aimed at the pursuit of the defined objective/purpose/goal and, in this way, generating modalities belonging to the generative class and of maximum generative impact. |
| **Cause of Action – CA**  (Stabilisation) | Discursive modality that configures reality through empirical-factual connections of cause-effects with value of truth, which determine an immutable course of events. The argumentation is not epistemologically founded. |
| **Confirmation – CP**  (Hybrid) | Discursive modality that configures reality by validating and supporting what expressed through the Repertory to which it relates. |
| **IV level** | |
| **Contraposition – CT**  (Stabilisation) | Discursive modality that configures reality through parallelism between two or more discourse’s parts, which are connected in terms that one excludes the other. The criteria that allow exclusion are not made explicit. |
| **Implication – IP**  (Hybrid) | Discursive modality that configures reality shaping the narrator's own and exclusive position regarding probable situations that could occur and that have not yet occurred, through a cause-effect rhetorical argumentative link. Those situations are reported in a tense (and time) following the one related to the main action (present perfect-simple past or present or future, present-future, etc.). |
| **Judgement – JM**  (Stabilisation) | Discursive modality that configures reality according to CR’s processual properties by using moral and/or qualitative attributes without making explicit the criteria used, shaping the narrator's own and exclusive reality which therefore is not shareable. |
| **Prediction – PV**  (Stabilisation) | Discursive modality that configures realities defining/stating a future scenario as a certain result of the development of a current scenario through a cause-effect rhetorical argumentative link. |
| **Justification – JT**  (Stabilisation) | Discursive modality that configures reality by entailing Stabilisation of the “current state of things”: it associates a situation to a previous one in order to legitimize a “state of things”, obstructing the use of other ways to handle or change what is happening. |
| **Non-Answer – NA**  (Stabilisation) | Discursive modality that configures reality in order to avoid the asked question - according to CR’s processual properties - establishing a “state of things” in which the narrator does not adhere properly to the process introduced by the question itself. |
| **Comment – CM**  (Stabilisation) | Discursive modality that configures reality in an inappropriate and irrelevant way to what is asked in the question following the narrator’s own and exclusive criteria, which are neither made explicit nor sharable. The argumentation does not allow to answer the question asked and it uses CR’s processual properties. |
| **Generalization – GE**  (Stabilisation) | Discursive modality that configures reality by responding inadequately to the question asked and using cross-context argumentations, thus not covering what is required. The criteria used are not epistemologically founded. |
| **Evaluation – EU**  (Hybrid) | Discursive modality that configures reality by stating a “state of things” funded on the narrator’s own and exclusive criteria, which, although explicit, are non-sharable. |
| **Declaration of Aims – DA** (Hybrid) | Discursive modality that configures reality by transposing the object of the request in a future perspective, without elements of certainty and probability as foundation. |
| **Proposal – PP**  (Generative) | Discursive modality that configures uncertain reality, possible in an achievable way and aimed at handling what is requested/offered according to TG’s processual properties. |
| **Delegating to others - DE** (Stabilisation) | Discursive mode that configures reality by delegating to third parties processes that are proper and exclusive to the narrator. |
| **V level** | |
| **Prescription – PT**  (Hybrid) | Discursive modality that configures reality as orders/directions given by a third “point of view” position compared to the narrator’s one. Establishes rules and/or objectives and/or roles to follow, in terms of what one “has to do” or “has not to do”. The argumentation acquires a structure founded on a relation of necessity set by a part of the text. |
| **Reshaping – RS**  (Hybrid) | Discursive modality that configures realities that limit the generative potential of what the configuration offers. The argumentation's reference is third and not referable to the narrator. |
| **Consideration – CS**  (Generative) | Discursive modality that configures reality by proposing an argument which uses criteria of analysis that can be shared among several interlocutors, namely that do not belong to any narrators exclusively, but need all of their contribution to maintain them (the criteria). |
| **VI level** | |
| **Anticipation – AT**  (Generative) | Discursive modality that configures reality through an argumentation shaped according to CS’s processual properties. This Repertory configures many different and uncertain situations that can occur and that have not yet occurred using PS’s processual properties. |

**Supplementary Material 4a – Discursive Repertories Distribution: Investigation Area 1**


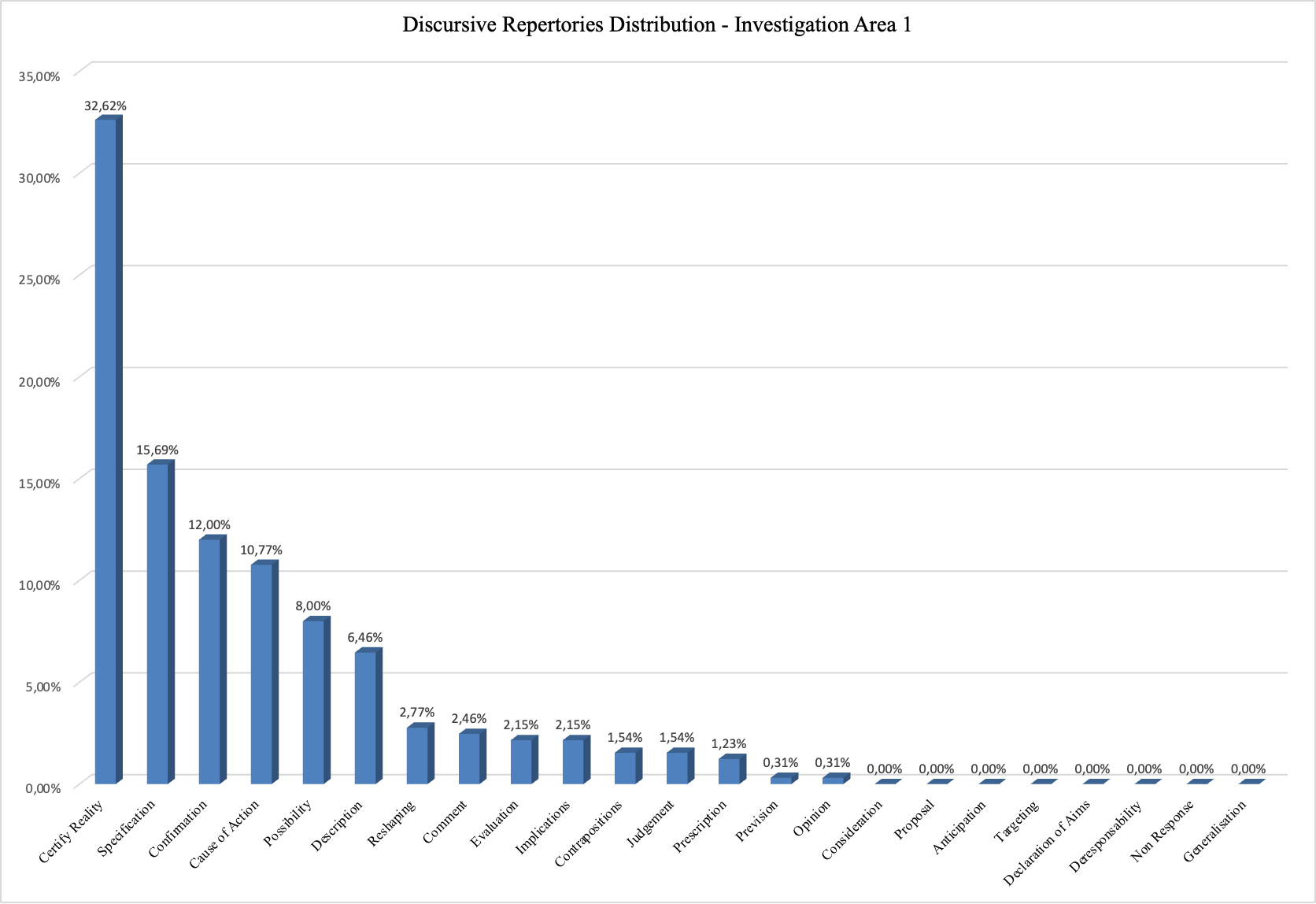


**Supplementary Material 4b – Discursive Repertories Distribution: Investigation Area 2**


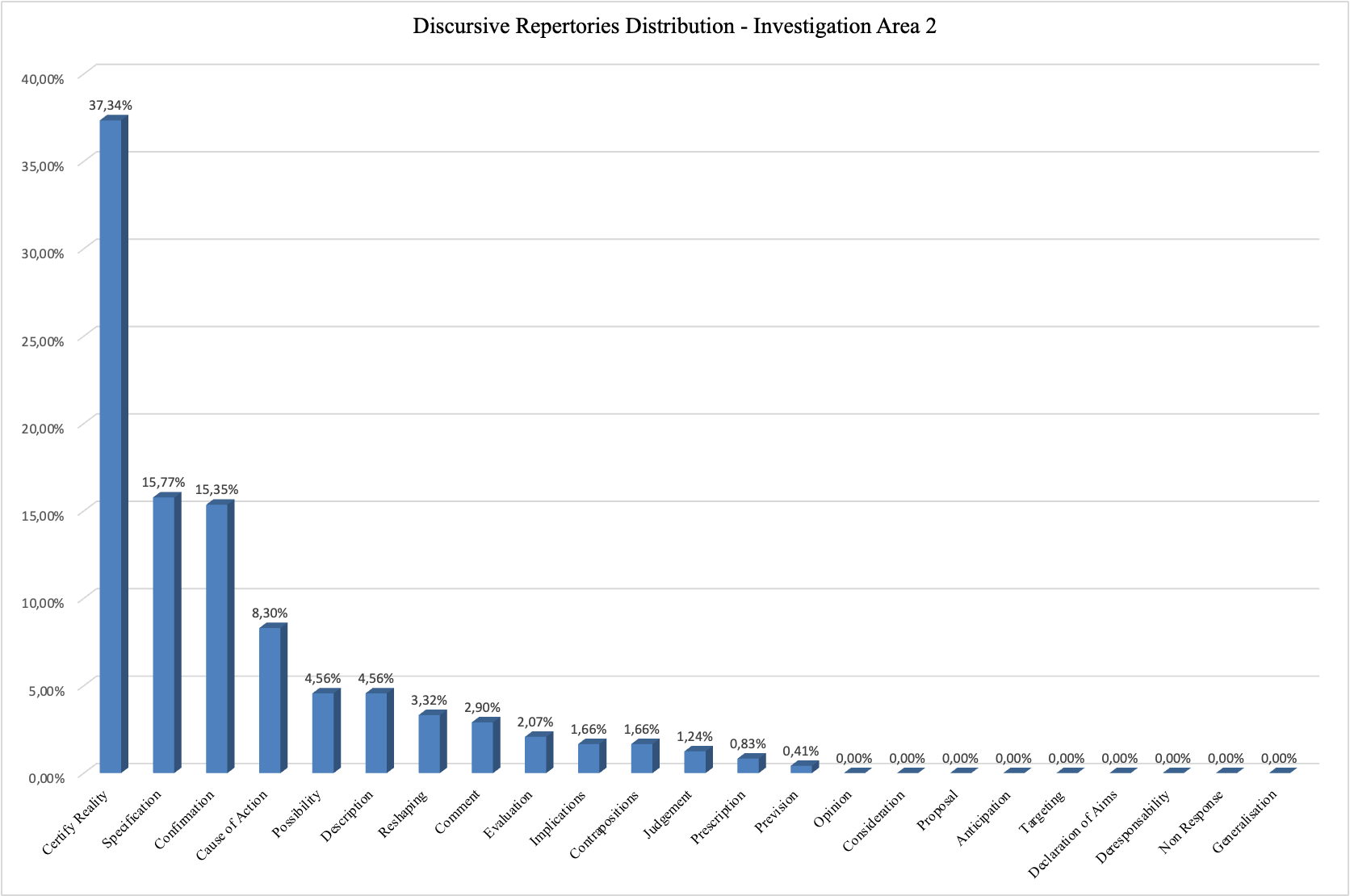


**Supplementary Material 4c – Discursive Repertories Distribution: Investigation Area 3**


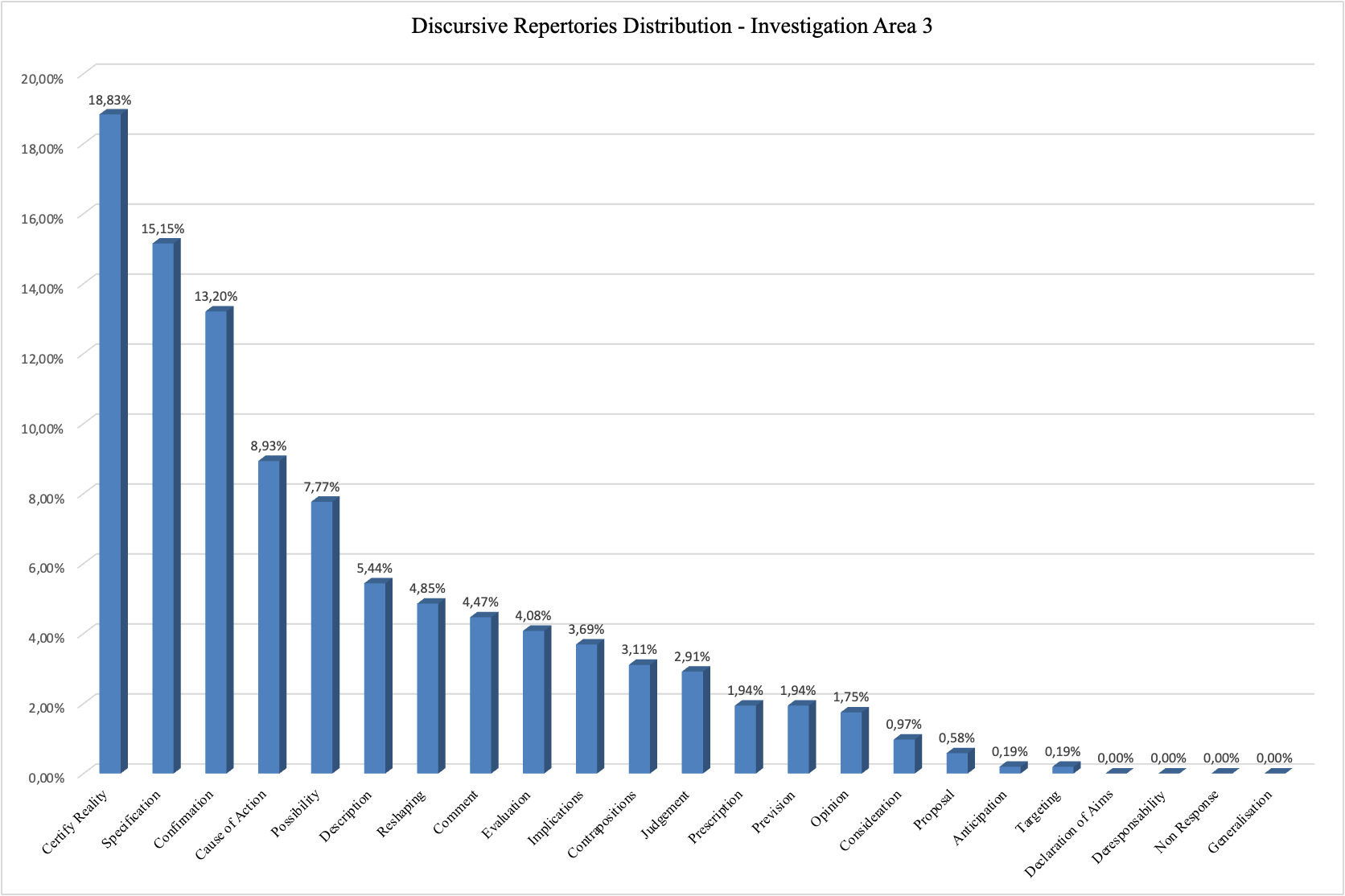


**Supplementary Material 4d – Discursive Repertories Distribution: Global Configuration**


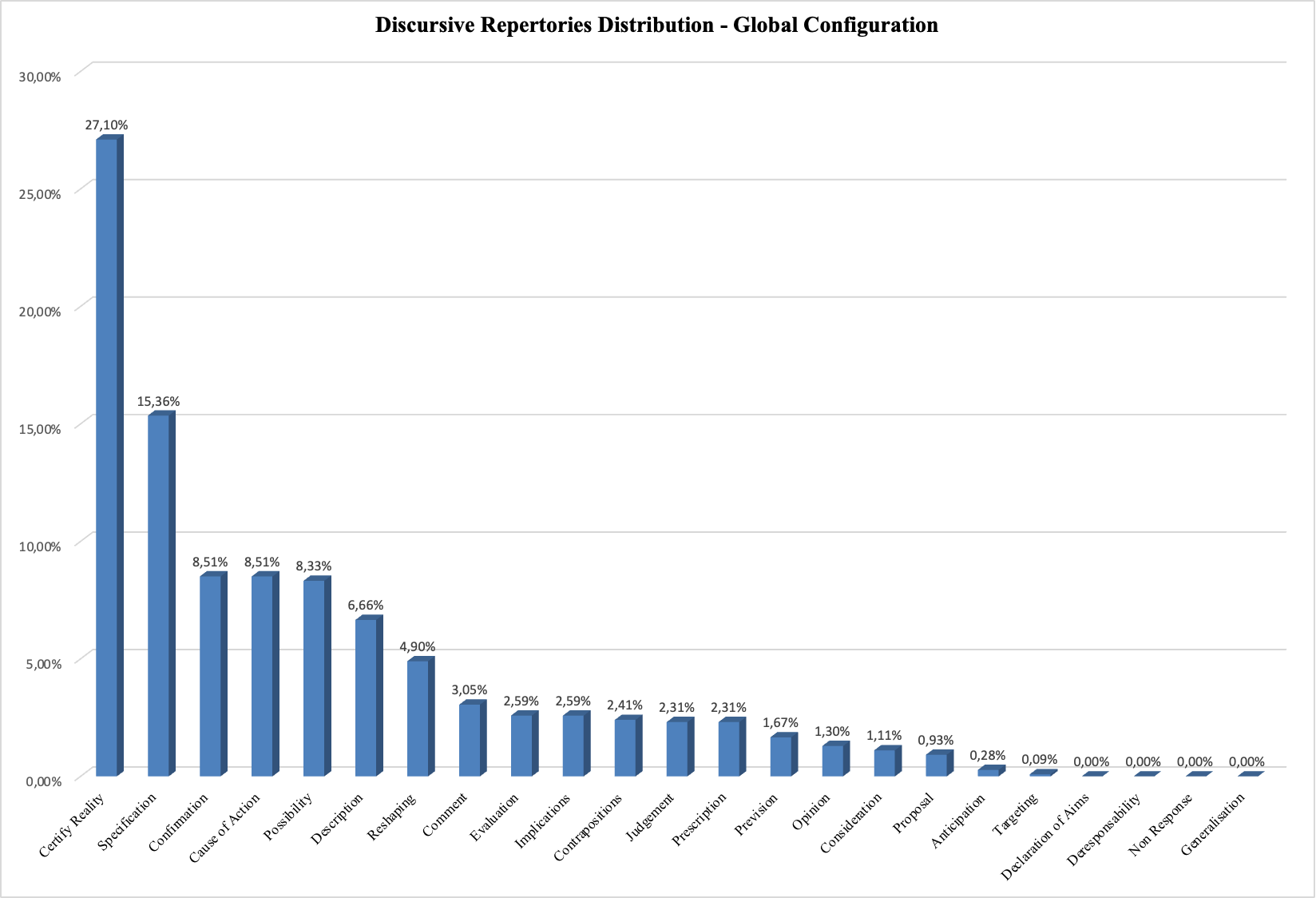

Supplement: Supplementary file 1 — Supplementary Material 1. [file 40359_2024_1767_MOESM1_ESM.docx]
